# Lumbar and Thoracolumbar Curves Are Associated with Coronal Lower Limb Malalignment in Adolescent Idiopathic Scoliosis

**DOI:** 10.3390/medicina62050978

**Published:** 2026-05-17

**Authors:** Ahmet Serhat Aydin, Emre Kocazeybek, Ahmet Mücteba Yildirim, Onur Kutlu, Serkan Bayram, Turgut Akgul

**Affiliations:** 1 Department of Orthopedics and Traumatology, School of Medicine, Akdeniz University, 07070 Antalya, Turkey; 2 Department of Orthopedics and Traumatology, Haseki Training and Research Hospital, 54600 Istanbul, Turkey; emrekcz@gmail.com; 3 Department of Orthopedics and Traumatology, Istanbul Medeniyet University, 34700 Istanbul, Turkey; ahmetmuctebaitf@gmail.com; 4 Department of Orthopedics and Traumatology, Igdir Training and Research Hospital, 76000 Igdir, Turkey; okorto93@gmail.com; 5 Department of Orthopedics and Traumatology, Istanbul Faculty of Medicine, Istanbul University, 34452 Istanbul, Turkey; dr.serkanbayram89@gmail.com (S.B.); trgtakgul@gmail.com (T.A.)

**Keywords:** scoliosis, adolescent, lower extremity, pelvis, radiography, biomechanical phenomena, leg length inequality

## Abstract

*Background and Objectives*: Adolescent idiopathic scoliosis (AIS) may influence pelvic orientation and lower-limb alignment; however, data on coronal lower-limb alignment after completion of spinal treatment remain limited. This study aimed to evaluate lower-limb radiographic alignment in AIS patients after spinal treatment and to determine whether these parameters differ according to main curve location. *Materials and Methods*: In this retrospective study, 70 AIS patients treated surgically (*n* = 52) or with brace therapy (*n* = 18) between 2010 and 2020 were analyzed. Patients were grouped according to main curve location as thoracic (*n* = 28), lumbar (*n* = 21), or thoracolumbar (*n* = 21). Pre-treatment standing full-spine radiographs were used to assess Cobb angle, coronal balance, and pelvic coronal obliquity angle (PCOA). After completion of spinal treatment, full-length weight-bearing lower-limb radiographs were evaluated for femoral and tibial lengths, mechanical axis deviation (MAD), femoral neck–shaft angle (NSA), anatomical lateral distal femoral angle (aLDFA), and mechanical lateral distal femoral angle (mLDFA). Additional treatment-stratified, treatment-adjusted, and threshold-based analyses were performed. *Results*: PCOA, coronal balance, bilateral MAD, right aLDFA, and right mLDFA differed significantly among the three curve-location groups. The lumbar group demonstrated more negative MAD values than the thoracic group, indicating a tendency toward valgus alignment (right MAD: −5.88 ± 8.8 mm vs. 3.65 ± 7.9 mm, *p* = 0.004; left MAD: −3.5 ± 7.5 mm vs. 3.75 ± 7.0 mm, *p* = 0.005). After adjustment for treatment modality, age, and main Cobb angle, curve location remained significantly associated with right MAD, left MAD, right aLDFA, and right mLDFA. However, the proportion of patients with clinically relevant malalignment, defined as MAD exceeding ±10 mm in at least one limb, did not differ significantly among the groups. *Conclusions*: AIS patients show subtle but measurable differences in coronal lower-limb alignment after completion of spinal treatment. Lumbar and thoracolumbar curves are associated with greater pelvic obliquity and a tendency toward more valgus mechanical-axis alignment, whereas limb lengths and NSA remain comparable among curve-location groups. These findings appear to represent mainly radiographic or biomechanical variations rather than overt clinically relevant deformity in most patients.

## 1. Introduction

Adolescent idiopathic scoliosis (AIS) is a three-dimensional spinal deformity that primarily affects the frontal plane and accounts for the majority of scoliosis cases diagnosed during adolescence [1,2]. Although AIS is classically considered a spinal disorder, it may also alter trunk balance, pelvic orientation, and compensatory lower-limb biomechanics [3,4,5]. Previous studies have shown that functional impairments may extend beyond the spine, including reduced upper-extremity function on the concave side of the curve and altered hip motion, particularly limitation of hip adduction on the dominant side [6,7].

With regard to the lower limbs, functional leg length discrepancy and pelvic obliquity have been reported in AIS, especially in patients with curves involving the lumbar region [8,9,10,11]. Distinguishing functional from structural leg length discrepancy is clinically important because functional inequality may reflect compensatory pelvic tilt, coronal imbalance, or postural adaptation rather than true bony shortening. This distinction is relevant when interpreting lower-limb radiographic parameters such as mechanical axis deviation (MAD), femoral and tibial length, and coronal femoral alignment. However, it remains unclear whether AIS is associated with consistent structural changes in lower-extremity morphology or whether the observed differences primarily represent functional or compensatory alignment changes related to curve pattern. Studies assessing proximal femoral morphology in AIS have also yielded conflicting results; some authors have reported increased femoral neck–shaft angles, whereas others have found lower values compared with earlier series [3,12,13].

Most previous investigations have focused on patients during the growth phase, while data on lower-limb morphology and coronal alignment after completion of spinal treatment remain limited [3,14,15,16]. Normal lower-limb alignment changes during growth and adolescence, and pediatric radiographic reference data have been established to define physiological anteroposterior lower-limb alignment parameters in children [17]. It has also been suggested that most lower-limb biomechanical development is largely completed before or during early adolescence, implying that later spinal deformity may have only limited influence on mature lower-limb morphology [14,15,16,17]. Nevertheless, long-standing coronal imbalance and pelvic obliquity may leave residual effects on lower-limb mechanical alignment, particularly in patients with lumbar or thoracolumbar curve patterns [8,9,10,11,18].

Accordingly, we hypothesized that coronal lower-limb alignment may remain altered after completion of spinal treatment in AIS and that these alterations may be associated with the location of the main spinal curve. The primary aim of this study was to investigate lower-extremity morphology and coronal alignment in AIS patients after completion of spinal treatment. The secondary aim was to evaluate whether lower-limb biomechanical parameters differ according to main curve location, classified as thoracic, lumbar, or thoracolumbar.

## 2. Materials and Methods

### 2.1. Study Design and Ethical Approval

This retrospective observational study included consecutive patients with adolescent idiopathic scoliosis (AIS) who were treated at a single tertiary referral center between January 2010 and December 2020. The study protocol was approved by the institutional ethics committee (8 February 2022, 2022/910), and all procedures were conducted in accordance with the Declaration of Helsinki. Written informed consent for the use of clinical and radiographic data was obtained from all patients and/or their legal guardians at the time of treatment.

### 2.2. Patient Selection

All patients who were diagnosed with AIS and treated either surgically or with brace therapy during the study period were screened for eligibility. Inclusion criteria were as follows: diagnosis of AIS according to Scoliosis Research Society criteria; age between 13 and 30 years at the time of final radiographic evaluation; completion of spinal treatment, defined as posterior spinal fusion or completed brace treatment with no further planned spinal intervention; and availability of standardized standing full-spine radiographs at initial presentation and full-length weight-bearing lower-limb radiographs obtained after completion of spinal treatment.

For surgically treated patients, completion of spinal treatment was defined as the postoperative stage after posterior spinal fusion when no additional spinal procedure was planned, and final post-treatment radiographs were available. For patients treated with brace therapy, completion of spinal treatment was defined as discontinuation of brace treatment after clinical follow-up and no further planned intervention. Post-treatment lower-limb radiographs were obtained at the final post-treatment evaluation after completion of surgical or brace management.

Exclusion criteria were as follows: history of lower-extremity surgery related to tumor, avascular necrosis, trauma, or deformity correction; revision posterior instrumentation; neuromuscular or syndromic scoliosis or other neurological disorders affecting the lower limbs; developmental dysplasia of the hip or other primary hip pathology; incomplete imaging; inadequate radiograph quality; or loss to follow-up.

**Figure 1:**
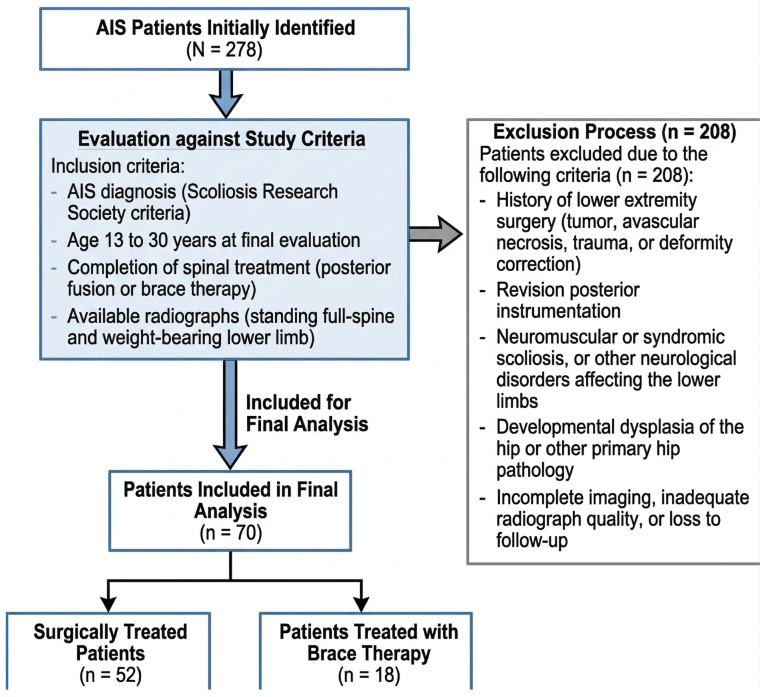
Flowchart demonstrating the study design, including patient selection, grouping according to curve location (thoracic, lumbar, and thoracolumbar), and the sequence of radiographic measurements.

Among 278 AIS patients initially identified, 208 were excluded according to these criteria, leaving 70 patients for the final analysis. Of these, 52 patients were treated surgically and 18 with brace therapy. The patient selection process is summarized in Figure 1.

### 2.3. Curve Classification and Grouping

All curves were classified using the Lenke classification system based on pre-treatment standing posteroanterior and lateral full-spine radiographs [19]. For the purposes of this study, patients were grouped according to the location of the main coronal curve apex and the corresponding Lenke curve pattern as follows:

Thoracic group: patients with a main thoracic curve pattern, including Lenke type 1 or 2 curves, *n* = 28.

Lumbar group: patients with a main thoracolumbar/lumbar curve pattern, including Lenke type 5 or 6 curves, *n* = 21.

Thoracolumbar group: patients with double-curve patterns involving both thoracic and thoracolumbar/lumbar components, including Lenke type 3 or 4 curves, *n* = 21.

The main Cobb angle was defined as the largest measured Cobb angle on pre-treatment standing full-spine radiographs and was used in baseline group comparisons and adjusted analyses.

### 2.4. Radiographic Acquisition

**Figure 2:**
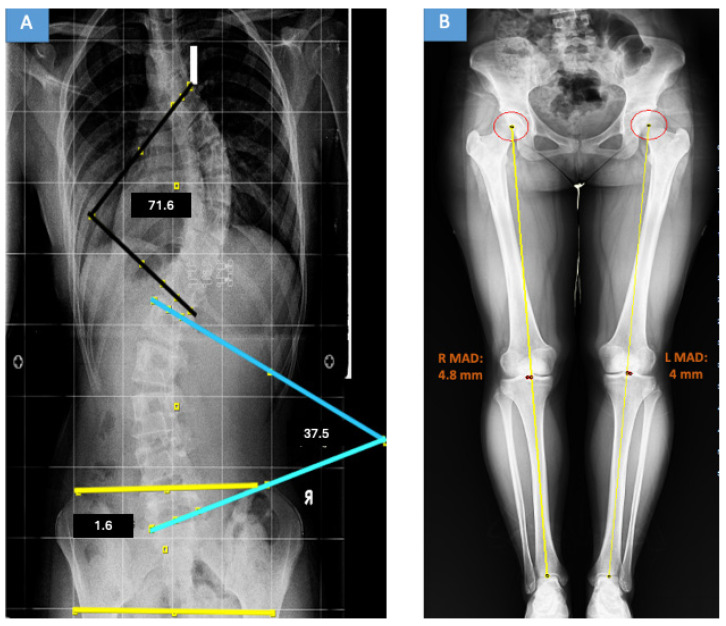
Representative radiographs of a patient with thoracic idiopathic scoliosis. (**A**) Pre-treatment standing full-spine anteroposterior radiograph demonstrating a Lenke type 3C curve pattern and pelvic obliquity. The colored lines indicate Cobb angle and pelvic obliquity measurements. (**B**) Post-treatment full-length weight-bearing lower-limb radiograph demonstrating mechanical-axis assessment; yellow lines indicate the mechanical axes, and marked points indicate reference landmarks for MAD measurement.

**Figure 3:**
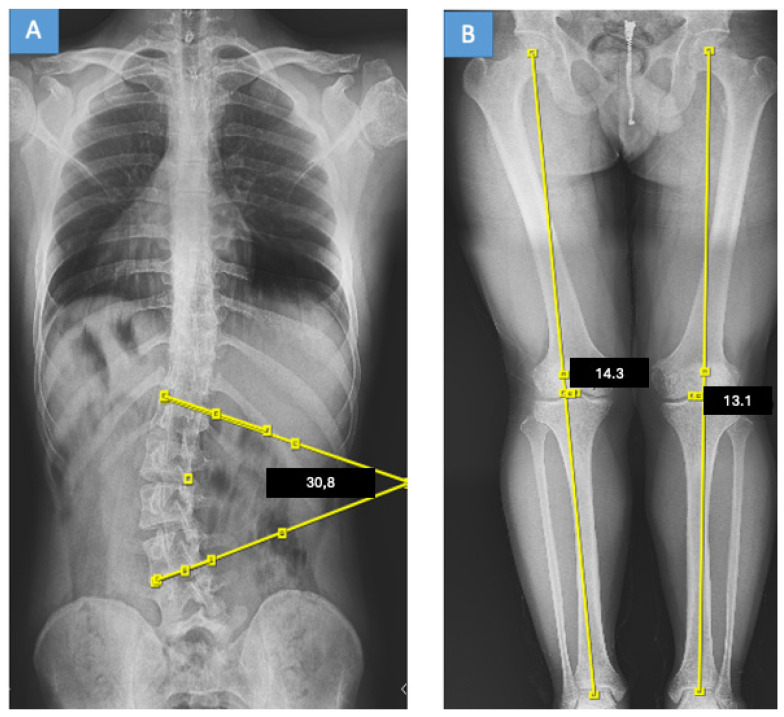
Representative radiographs of a patient with lumbar idiopathic scoliosis and a Lenke type 5C curve treated non-operatively with bracing. (**A**) Standing full-spine anteroposterior radiograph showing pelvic obliquity. (**B**) Full-length weight-bearing lower-limb radiograph showing mechanical-axis assessment; yellow lines indicate the mechanical axes, and marked points indicate reference landmarks for MAD measurement.

At initial presentation, all patients underwent standard standing full-spine posteroanterior and lateral radiographs, including the pelvis. These radiographs were used to determine Cobb angles, coronal balance, pelvic coronal obliquity angle (PCOA), and Lenke classification (Figure 2A and Figure 3A).

After completion of spinal treatment, patients were re-evaluated using standardized full-length weight-bearing lower-limb radiographs. Radiographs were obtained with the patellae facing directly forward and the knees in full extension to minimize rotational variation during assessment of coronal lower-limb alignment (Figure 2B and Figure 3B). These radiographs were acquired after approval of the institutional ethics committee.

### 2.5. Radiographic Measurements

Spinal parameters were measured on standing full-spine radiographs. Cobb angles of structural curves were recorded. Coronal balance was defined as the horizontal distance between the C7 plumb line and the central sacral vertical line. PCOA was defined as the angle between a line connecting the most superior points of both iliac crests and a true horizontal reference line [9,10].

On full-length weight-bearing lower-limb radiographs, the following parameters were evaluated bilaterally:

Femoral length: distance from the superior aspect of the femoral head to the distal medial femoral condyle.

Tibial length: distance from the proximal tibial joint line to the distal articular surface of the tibial plafond.

Mechanical axis deviation (MAD): perpendicular distance in millimeters from the mechanical axis line, defined as the line connecting the center of the femoral head to the center of the ankle joint, to the center of the knee joint. Negative values indicated valgus alignment, whereas positive values indicated varus alignment [3,12,13].

Femoral neck–shaft angle (NSA): angle between the axis of the femoral neck and the longitudinal axis of the femoral shaft, corresponding to the standard femoral neck–shaft, or caput-collum-diaphyseal, angle [3,12,13].

Mechanical lateral distal femoral angle (mLDFA): the lateral angle between the mechanical axis of the femur and the distal femoral joint line.

Mechanical lateral proximal femoral angle (mLPFA): the lateral angle between the mechanical axis of the femur and the proximal femoral joint line.

Anatomical lateral distal femoral angle (aLDFA): the lateral angle between the anatomical axis of the femur and the distal femoral joint line.

Anatomical medial proximal femoral angle (aMPFA): the medial angle between the anatomical axis of the femur and the proximal femoral joint line.

All radiographic measurements were performed using a picture archiving and communication system workstation (Extreme PACS, Ankara, Turkey). Two senior orthopedic surgeons independently measured all parameters twice, with at least a 1-week interval between the two measurement sessions. For the main analysis, the mean of the four measurements was used.

Intraobserver and interobserver reliability were assessed for the main radiographic measurement parameters, including PCOA, MAD, aLDFA, and mLDFA. Reliability was calculated using a two-way random-effects intraclass correlation coefficient model with absolute agreement and 95% confidence intervals. For interobserver reliability, the mean values of the two repeated measurements obtained by each observer were compared. ICC values were interpreted as poor (<0.50), moderate (0.50–0.75), good (0.75–0.90), and excellent (>0.90).

### 2.6. Statistical Analysis

Statistical analyses were performed using IBM SPSS Statistics version 24.0 (IBM Corp., Armonk, NY, USA). Descriptive statistics were expressed as mean ± standard deviation, minimum and maximum values for continuous variables, and as frequencies and percentages for categorical variables. The Shapiro–Wilk test was used to assess the normality of continuous variables.

Baseline demographic and clinical characteristics, including age, sex, treatment modality, Risser sign, Lenke type, and main Cobb angle, were compared among the thoracic, lumbar, and thoracolumbar curve-location groups. Continuous variables were compared using one-way analysis of variance for normally distributed variables. Bonferroni post hoc tests were used for pairwise comparisons when appropriate. Categorical variables were compared using the chi-square test or Fisher’s exact/Monte Carlo exact test where appropriate.

The primary analysis compared radiographic parameters among the three curve-location groups. These parameters included PCOA, coronal balance, femoral and tibial lengths, MAD, NSA, aLDFA, mLDFA, aMPFA, and mLPFA. Because treatment modality could potentially influence pelvic and lower-limb alignment, additional stratified analyses were performed separately in surgically treated and brace-treated patients.

To further address the potential confounding effect of treatment modality, adjusted regression models were constructed for the main radiographic outcomes, including PCOA, right and left MAD, right and left aLDFA, and right and left mLDFA. In the first model, curve location and treatment modality were included as independent variables. In the second model, age and the main Cobb angle were added as additional covariates.

To evaluate the clinical relevance of statistically significant MAD differences, an additional threshold-based subgroup analysis was performed. A MAD threshold of ±10 mm was used to identify clinically relevant mechanical-axis deviation. Patients were categorized as having valgus deviation below −10 mm, alignment between −10 and +10 mm, or varus deviation above +10 mm for each limb. The proportion of patients with MAD exceeding ±10 mm in either limb was compared among curve-location groups.

Statistical significance was set at *p* < 0.05. With the available sample size, the study was considered adequately powered to detect large between-group differences, whereas smaller effects may have remained undetected.

## 3. Results

Among 278 patients with adolescent idiopathic scoliosis who were screened during the study period, 208 were excluded according to the predefined exclusion criteria. The most common reasons for exclusion were missing or inadequate lower-limb radiographs (*n* = 72), loss to follow-up (*n* = 58), neuromuscular or syndromic scoliosis (*n* = 29), lower-extremity surgery related to tumor, trauma, or deformity correction (*n* = 15), developmental dysplasia of the hip (*n* = 14), revision posterior instrumentation (*n* = 12), and other primary hip pathology (*n* = 8). The remaining 70 patients constituted the final study cohort. Of these, 52 patients had been treated surgically and 18 had completed brace treatment. The patient selection process is summarized in Figure 1.

Based on the location of the main coronal curve, 28 patients were included in the thoracic group, 21 in the lumbar group, and 21 in the thoracolumbar group. Baseline demographic and clinical characteristics of the three curve-location groups are presented in Table 1. There were no statistically significant differences among the groups in terms of age, sex distribution, treatment modality, Risser sign, or main Cobb angle.

For the entire cohort, the mean pelvic coronal obliquity angle (PCOA) was 2.3 ± 1.9° (range, 0–12°), and the mean coronal balance was −1.14 ± 1.57 cm (range, −4.6 to 3.0 cm). The mean right femoral length was 479 ± 37 mm, and the mean left femoral length was 479 ± 37 mm. The mean right tibial length was 391 ± 32 mm, and the mean left tibial length was 392 ± 32 mm. The mean right mechanical axis deviation (MAD) was −0.41 ± 10.2 mm, and the mean left MAD was −0.7 ± 8.0 mm. The mean right and left femoral neck–shaft angles (NSA) were 133 ± 4° and 133 ± 5°, respectively. Detailed radiographic parameters according to curve-location group are presented in Table 2.

Significant differences among the three groups were found for PCOA, coronal balance, bilateral MAD, right aLDFA, and right mLDFA. Post hoc analysis showed that PCOA and coronal balance were significantly different between the thoracolumbar and thoracic groups (*p* = 0.011 and *p* = 0.004, respectively), whereas no significant differences were observed between the thoracic and lumbar groups or between the lumbar and thoracolumbar groups for these parameters.

For MAD, significant differences were observed between the lumbar and thoracic groups for both limbs (right MAD, *p* = 0.004; left MAD, *p* = 0.005). The lumbar group demonstrated bilateral valgus alignment, with mean right MAD of −5.88 ± 8.8 mm and mean left MAD of −3.5 ± 7.5 mm. No significant differences were found between the thoracic and thoracolumbar groups or between the lumbar and thoracolumbar groups for MAD.

Regarding distal femoral alignment, the thoracic group had significantly higher right aLDFA and right mLDFA than both the lumbar group (*p* = 0.026 and *p* = 0.022, respectively) and the thoracolumbar group (*p* = 0.014 and *p* = 0.006, respectively). No significant differences were observed between the lumbar and thoracolumbar groups for these parameters. No significant between-group differences were found in femoral length, tibial length, NSA, aMPFA, or mLPFA on either side.

Intraobserver and interobserver reliability values for the main radiographic measurements are presented in Table 3.

Interobserver ICC values ranged from 0.872 to 0.934, indicating good-to-excellent agreement for PCOA, MAD, aLDFA, and mLDFA measurements. Intraobserver reliability was also generally good to excellent, with ICC values ranging from 0.763 to 0.913 for most parameters; only the Observer 2 intraobserver ICC for right MAD was moderate but close to the threshold for good reliability.

To address the potential confounding effect of treatment modality, additional stratified and adjusted analyses were performed. In the stratified analysis, curve-location-related differences in MAD and right-sided distal femoral angles were observed in both surgically treated and brace-treated patients, although the brace-treated subgroup was small, particularly for thoracolumbar curves. In treatment-adjusted regression models, curve location remained significantly associated with right MAD, left MAD, right aLDFA, and right mLDFA. These associations also remained significant after further adjustment for age and main Cobb angle. By contrast, PCOA did not remain statistically significant after adjustment for treatment modality, age, and main Cobb angle. Treatment modality itself was not independently associated with the main radiographic outcomes in the adjusted models (Table 4).

To evaluate the clinical relevance of the statistically significant MAD differences, an additional threshold-based analysis was performed using ±10 mm as the cutoff for clinically relevant mechanical-axis deviation. Overall, 19 of 70 patients (27.1%) had MAD exceeding ±10 mm in at least one limb. The proportion of patients with MAD exceeding ±10 mm in any limb did not differ significantly among the thoracic, lumbar, and thoracolumbar groups (21.4%, 33.3%, and 28.6%, respectively; *p* = 0.641). However, the categorical distribution of right MAD differed significantly among groups (*p* = 0.017), mainly because the lumbar group had a higher frequency of right-sided valgus deviation below −10 mm. Left-sided MAD category distribution did not differ significantly among groups (*p* = 0.183) (Table 5).

## 4. Discussion

In this study, we evaluated coronal lower-limb morphology and alignment in patients with AIS after completion of spinal treatment and compared radiographic parameters according to main curve location. The principal findings were that pelvic coronal obliquity angle (PCOA), coronal balance, bilateral mechanical axis deviation (MAD), right anatomical lateral distal femoral angle (aLDFA), and right mechanical lateral distal femoral angle (mLDFA) differed significantly among curve-location groups. Lumbar curves were associated with more negative MAD values, indicating a tendency toward valgus mechanical-axis alignment, whereas femoral and tibial lengths and femoral neck–shaft angle (NSA) were comparable among groups. Additional treatment-adjusted analyses suggested that these differences were not primarily explained by treatment modality, while the threshold-based MAD analysis indicated that most of the observed differences were subtle radiographic or biomechanical variations rather than overt clinically relevant deformities.

Previous studies have shown that AIS may influence pelvic orientation and lower-limb biomechanics [3,4,5]. Saji et al. reported increased femoral neck–shaft angles in AIS patients compared with healthy controls, suggesting that proximal femoral morphology may adapt to spinal deformity [12]. In contrast, Markus et al. found lower collodiaphyseal angles and altered lower-limb biomechanical parameters in AIS, highlighting discrepancies among AIS cohorts and measurement techniques [13]. In the present study, NSA values were similar among the three curve-location groups and remained within commonly reported reference ranges. These findings suggest that, after completion of spinal treatment, the location of the main spinal curve may have a limited influence on proximal femoral morphology.

The distinction between functional and structural lower-limb discrepancy is important in AIS. Sekiya et al. showed that AIS patients frequently demonstrate functional leg length discrepancy, whereas true structural discrepancy is less prominent [8]. This concept is supported by our findings, as femoral and tibial lengths did not differ significantly among curve-location groups. Therefore, the lower-limb alignment differences observed in this cohort appear to be more closely related to pelvic tilt, coronal imbalance, and compensatory mechanical-axis adaptation than to true bony length discrepancy.

Pelvic obliquity represents an important biomechanical link between spinal deformity and lower-limb alignment [8,9,10,11,18]. Cho et al. and Ploumis et al. reported associations between pelvic obliquity, coronal imbalance, and AIS curve characteristics [9,11]. Chan et al. further demonstrated that pelvic obliquity is more frequently observed in patients with distal curve patterns, particularly Lenke 5 and 6 curves, than in those with thoracic curves [10]. In the present cohort, PCOA and coronal balance were significantly greater in thoracolumbar curves than in thoracic curves, supporting the concept that distal curve patterns are more closely related to pelvic coronal malalignment. However, PCOA did not remain statistically significant after adjustment for treatment modality, age, and main Cobb angle, suggesting that pelvic obliquity may be influenced by multiple interacting factors rather than curve location alone.

The magnitude of pelvic alignment changes observed in this study should also be interpreted cautiously. Recent quantitative evidence from hip-preservation surgery has shown that pelvic tilt remains largely unchanged after periacetabular osteotomy, with only minimal and non-significant postoperative shifts [20]. Although this evidence comes from a different patient population, it supports the broader concept that pelvic orientation may be relatively resistant to large structural changes after skeletal maturation or corrective intervention. Therefore, the pelvic obliquity differences observed in AIS patients may represent subtle residual coronal compensation rather than major structural pelvic remodeling.

MAD is a clinically relevant parameter because it reflects the overall coronal mechanical axis of the lower limb and may influence load distribution across the knee. In our study, the lumbar group demonstrated more negative MAD values than the thoracic group bilaterally, indicating a tendency toward valgus alignment. However, the absolute mean differences were small. To better clarify clinical relevance, we performed an additional threshold-based analysis using ±10 mm as a cutoff for clinically relevant mechanical-axis deviation. Although right-sided MAD category distribution differed among groups, the proportion of patients with MAD exceeding ±10 mm in at least one limb did not differ significantly among the thoracic, lumbar, and thoracolumbar groups. Thus, the statistically significant group-level MAD differences should be interpreted primarily as subtle radiographic or biomechanical findings rather than clinically obvious malalignment in most patients.

Because an internal healthy control group was not available, we contextualized the present findings using published reference values for lower-limb alignment parameters. This comparison is summarized in Table 6. Overall, the mean MAD values in the present cohort were close to neutral alignment, while NSA and mLDFA values were within published reference ranges. Mean aLDFA values were slightly above the upper reference limit, but the magnitude of this difference was small and should be interpreted cautiously because lower-limb alignment parameters vary according to age, sex, skeletal maturity, ethnicity, radiographic technique, and measurement method.

The heterogeneity introduced by different treatment modalities is another important consideration. In the present cohort, 52 patients were treated surgically and 18 with brace therapy. Surgical correction may influence coronal balance and pelvic orientation, whereas brace treatment may be associated with different compensatory mechanisms. For this reason, we performed additional stratified and treatment-adjusted analyses. Curve location remained significantly associated with right MAD, left MAD, right aLDFA, and right mLDFA after adjustment for treatment modality, age, and main Cobb angle. Treatment modality itself was not independently associated with the main radiographic outcomes. These findings suggest that the observed lower-limb alignment differences were not primarily driven by surgical versus brace treatment. Nevertheless, the brace-treated subgroup was relatively small, particularly in the thoracolumbar group, and the stratified findings should therefore be interpreted as exploratory.

Reliable measurement of coronal lower-limb alignment is essential when interpreting small differences in MAD and distal femoral angles. In the present study, intraobserver and interobserver ICC values demonstrated good-to-excellent reliability for the main radiographic parameters, supporting the reproducibility of the measurement protocol. Recent evidence has shown that artificial intelligence-guided assessment of the hip-knee-ankle angle on standing full-leg radiographs demonstrates excellent correlation with experienced human raters [24]. Although all measurements in the present study were performed manually by experienced orthopedic surgeons, this emerging evidence emphasizes the importance of standardized, reproducible, and potentially automated measurement protocols when evaluating subtle coronal alignment differences.

Rehabilitation protocols and functional gait recovery may also influence the clinical interpretation of subtle radiographic alignment differences. Technology-assisted and mechatronic gait rehabilitation systems have been developed to support gait training in patients with ambulation disorders, and usability studies have emphasized the importance of patient-centered feedback, safety, comfort, and therapeutic efficiency in rehabilitation technologies [25]. The present study did not evaluate postoperative rehabilitation protocols, muscle function, or objective gait parameters. Future AIS studies should therefore integrate radiographic alignment assessment with standardized rehabilitation data, functional gait analysis, and patient-reported outcomes to determine whether subtle coronal alignment differences translate into clinically meaningful functional consequences.

This study has several limitations. First, its retrospective design and the requirement for standardized full-length lower-limb radiographs introduce potential selection bias. A large proportion of the initially screened cohort was excluded, mainly because of missing or inadequate lower-limb radiographs and loss to follow-up. This may have reduced the representativeness of the final cohort, as patients with complete and high-quality radiographic datasets may differ from excluded patients. Second, the study did not include an internal age-matched healthy control group. For ethical and practical reasons, obtaining full-length weight-bearing radiographs from healthy adolescents was not feasible; therefore, the findings were interpreted using published reference values. Third, the sample size was limited, particularly for subgroup and stratified analyses. The study was likely sufficient to detect large between-group differences, but smaller effects may have remained undetected. Fourth, sagittal plane parameters, three-dimensional pelvic morphology, muscle function, rehabilitation protocols, and gait parameters were not assessed. Finally, the study included both surgically treated and brace-treated patients, and although adjusted analyses were performed, residual confounding related to treatment type cannot be completely excluded.

Despite these limitations, this study adds to the limited literature on lower-limb alignment after completion of spinal treatment in AIS. The findings suggest that distal curve patterns, particularly lumbar and thoracolumbar curves, are associated with subtle differences in pelvic obliquity and coronal mechanical-axis alignment. However, these differences appear to be mostly radiographic or biomechanical rather than overtly clinically relevant in most patients. Future prospective multicenter studies with matched control groups, standardized rehabilitation data, three-dimensional imaging, and objective gait analysis are needed to better clarify the relationship between spinal curvature, pelvic compensation, and lower-limb biomechanics in AIS.

## 5. Conclusions

Adolescent idiopathic scoliosis may be associated with subtle differences in coronal lower-limb alignment after completion of spinal treatment. In this cohort, lumbar and thoracolumbar curves were associated with greater pelvic obliquity and more negative mechanical axis deviation, suggesting a tendency toward valgus alignment, whereas femoral and tibial lengths and femoral neck–shaft angles were comparable among groups.

Although some differences were statistically significant, their absolute magnitude was small, and clinically relevant mechanical-axis deviation exceeding ±10 mm did not differ among groups. Therefore, these findings should be interpreted mainly as subtle radiographic or biomechanical variations rather than overt lower-limb deformity, but distal curve patterns may still warrant attention during clinical follow-up.

## Figures and Tables

**Table 1:** Baseline demographic and clinical characteristics of the three curve-location groups.

Variable	Thoracic Scoliosis *n* = 28	Lumbar Scoliosis *n* = 21	Thoracolumbar Scoliosis *n* = 21	*p* Value
Age, years, mean ± SD, min–max	21.9 ± 3.3, 18–35	22.5 ± 3.8, 17–31	21.8 ± 3.8, 18–32	0.771
Sex, female/male	24/4	19/2	18/3	0.862
Treatment, surgery/brace	20/8	14/7	18/3	0.334
Risser sign, 2/3/4/5	3/8/14/3	1/6/10/4	2/4/12/3	0.934
Main Cobb angle, °, mean ± SD, min–max	52.6 ± 21.1, 23–105	40.9 ± 19.2, 15–100	45.5 ± 16.0, 25–90	0.108
Lenke type	1A: 10; 1B: 6; 1C: 3; 2C: 3; 3C: 6	5A: 1; 5B: 2; 5C: 14; 6C: 4	5C: 16; 6B: 1; 6C: 4	—

SD: standard deviation. The main Cobb angle was defined as the largest measured Cobb angle for each patient on pre-treatment standing full-spine radiographs. Age and main Cobb angle were compared using one-way analysis of variance. Sex, treatment modality, and Risser sign were compared using the chi-square test. Lenke type was presented descriptively because curve-location grouping was based on curve pattern.

**Table 2:** Radiographic parameters according to curve-location group.

Parameter	Thoracic Scoliosis *n* = 28	Lumbar Scoliosis *n* = 21	Thoracolumbar Scoliosis *n* = 21	*p* Value
Pelvic obliquity, °	1.57 ± 1.33, 0–4	2.67 ± 1.37, 0–5	3.15 ± 2.92, 0–12	0.032 *
Coronal balance, cm	−0.47 ± 1.4, −4.6–1.8	−1.54 ± 1.4, −4–2	−2.06 ± 1.1, −4–0	0.014 *
Right femoral length, mm	480 ± 46, 349–566	488 ± 29, 435–548	467 ± 32, 407–512	0.290
Left femoral length, mm	481 ± 45, 352–567	487 ± 29, 432–549	468 ± 30, 408–507	0.345
Right tibial length, mm	388 ± 39, 283–456	399 ± 23, 356–440	387 ± 31, 332–428	0.509
Left tibial length, mm	389 ± 40, 289–460	399 ± 22, 357–438	389 ± 32, 332–429	0.581
Right MAD, mm	3.65 ± 7.9, −8–20	−5.88 ± 8.8, −24–7	0.81 ± 12, −27–15	0.012 *
Left MAD, mm	3.75 ± 7.0, −10–16	−3.5 ± 7.5, −16–11	−1.07 ± 8.3, −11–13	0.015 *
Right NSA, °	132.6 ± 5.0, 128–150	133.2 ± 4.0, 124–141	133.6 ± 4.0, 126–139	0.816
Left NSA, °	132.1 ± 6.0, 122–150	134.2 ± 4.0, 125–143	134.6 ± 3.0, 128–139	0.294
Right aLDFA, °	84.7 ± 3.0, 79–92	82.6 ± 3.0, 77–89	82.2 ± 2.0, 79–87	0.023 *
Left aLDFA, °	84.2 ± 3.0, 80–93	83.3 ± 3.1, 77–92	82.8 ± 2.3, 79–88	0.374
Right mLDFA, °	90.4 ± 3.1, 85–98	88.1 ± 3.3, 82–94	87.4 ± 2.0, 84–93	0.012 *
Left mLDFA, °	89.6 ± 3.3, 84–99	88.9 ± 3.2, 83–99	88.2 ± 2.0, 85–93	0.457
Right aMPFA, °	86.4 ± 2.9, 81–95	88.6 ± 4.8, 77–99	87.8 ± 3.0, 77–92	0.093
Left aMPFA, °	87.0 ± 4.2, 74–97	87.6 ± 5.1, 76–101	88.6 ± 3.5, 76–93	0.456
Right mLPFA, °	87.3 ± 3.8, 75–93	86.2 ± 4.5, 78–97	86.5 ± 4.1, 81–96	0.731
Left mLPFA, °	86.5 ± 4.4, 77–99	86.5 ± 4.8, 76–98	86.0 ± 4.5, 80–98	0.946

Values are presented as mean ± standard deviation, minimum–maximum. MAD: mechanical axis deviation; NSA: neck–shaft angle; aLDFA: anatomical lateral distal femoral angle; mLDFA: mechanical lateral distal femoral angle; aMPFA: anatomical medial proximal femoral angle; mLPFA: mechanical lateral proximal femoral angle. * Statistically significant.

**Table 3:** Intraobserver and interobserver reliability of the main radiographic measurements.

Parameter	Observer 1 Intraobserver ICC	95% CI	Observer 2 Intraobserver ICC	95% CI	Interobserver ICC	95% CI	Reliability
PCOA	0.870	0.800–0.917	0.830	0.739–0.891	0.872	0.802–0.919	Good
Right MAD	0.854	0.775–0.907	0.735	0.605–0.827	0.880	0.814–0.924	Good
Left MAD	0.884	0.820–0.926	0.810	0.711–0.878	0.926	0.883–0.953	Excellent
Right aLDFA	0.913	0.864–0.945	0.857	0.779–0.908	0.934	0.896–0.958	Excellent
Left aLDFA	0.877	0.810–0.922	0.803	0.701–0.873	0.916	0.868–0.947	Excellent
Right mLDFA	0.904	0.850–0.939	0.810	0.711–0.878	0.930	0.890–0.956	Excellent
Left mLDFA	0.863	0.789–0.913	0.763	0.644–0.846	0.893	0.834–0.932	Good

ICC: intraclass correlation coefficient; CI: confidence interval; PCOA: pelvic coronal obliquity angle; MAD: mechanical axis deviation; aLDFA: anatomical lateral distal femoral angle; mLDFA: mechanical lateral distal femoral angle. ICC values were interpreted as poor (<0.50), moderate (0.50–0.75), good (0.75–0.90), and excellent (>0.90).

**Table 4:** Treatment-adjusted regression models for the main radiographic outcomes.

Parameter	Curve-Location *p* Value, Model 1	Treatment-Modality *p* Value, Model 1	Curve-Location *p* Value, Model 2	Treatment-Modality *p* Value, Model 2
PCOA	0.072	0.139	0.099	0.187
Right MAD	<0.001	0.575	0.001	0.484
Left MAD	<0.001	0.137	<0.001	0.056
Right aLDFA	0.001	0.699	0.003	0.765
Left aLDFA	0.145	0.716	0.180	0.697
Right mLDFA	<0.001	0.893	0.001	0.808
Left mLDFA	0.211	0.771	0.274	0.884

Model 1: adjusted for curve location and treatment modality. Model 2: adjusted for curve location, treatment modality, age, and main Cobb angle. PCOA: pelvic coronal obliquity angle; MAD: mechanical axis deviation; aLDFA: anatomical lateral distal femoral angle; mLDFA: mechanical lateral distal femoral angle.

**Table 5:** Threshold-based analysis of clinically relevant mechanical axis deviation according to curve-location group.

Curve-Location Group	*n*	Right MAD < −10 mm	Right |MAD| > 10 mm	Left MAD < −10 mm	Left |MAD| > 10 mm	Any Limb |MAD| > 10 mm
Thoracic	28	0 (0.0)	4 (14.3)	0 (0.0)	4 (14.3)	6 (21.4)
Lumbar	21	6 (28.6)	6 (28.6)	3 (14.3)	4 (19.0)	7 (33.3)
Thoracolumbar	21	2 (9.5)	5 (23.8)	1 (4.8)	2 (9.5)	6 (28.6)
*p* value	—	0.017	0.459	0.183	0.678	0.641

Values are presented as *n* (%). A threshold of ±10 mm was used to identify clinically relevant mechanical-axis deviation. Negative MAD values indicate valgus deviation, whereas positive values indicate varus deviation. MAD: mechanical axis deviation.

**Table 6:** Comparison of the present AIS cohort with published reference values for lower-limb alignment parameters.

Parameter	Present AIS Cohort	Published Reference Value/Range	Reference(s)	Interpretation
Right MAD	−0.41 ± 10.2 mm	Mechanical axis normally passes close to the knee center, commonly slightly medial; values within approximately ±10 mm are often considered close to neutral alignment	Paley and Tetsworth [21]; Paley [22]; Sabharwal et al. [23]; Popkov et al. [17]	Mean value close to neutral alignment
Left MAD	−0.70 ± 8.0 mm	Mechanical axis normally passes close to the knee center, commonly slightly medial; values within approximately ±10 mm are often considered close to neutral alignment	Paley and Tetsworth [21]; Paley [22]; Sabharwal et al. [23]; Popkov et al. [17]	Mean value close to neutral alignment
Right NSA	133 ± 4°	Approximately 130°; commonly cited normal range 124–136°	Paley [22]	Within published reference range
Left NSA	133 ± 5°	Approximately 130°; commonly cited normal range 124–136°	Paley [22]	Within published reference range
Right aLDFA	83.3 ± 4.5°	Approximately 81°; commonly cited normal range 79–83°	Paley [22]; Popkov et al. [17]	Slightly above the upper reference limit, but close to normal range
Left aLDFA	83.5 ± 2.9°	Approximately 81°; commonly cited normal range 79–83°	Paley [22]; Popkov et al. [17]	Slightly above the upper reference limit, but close to normal range
Right mLDFA	88.8 ± 3.1°	Approximately 87–88°; commonly cited normal range 85–90°	Paley [22]; Sabharwal et al. [23]; Popkov et al. [17]	Within published reference range
Left mLDFA	89.0 ± 2.9°	Approximately 87–88°; commonly cited normal range 85–90°	Paley [22]; Sabharwal et al. [23]; Popkov et al. [17]	Within published reference range

Values are presented as mean ± standard deviation. Negative MAD values indicate valgus deviation, whereas positive MAD values indicate varus deviation. Published reference values should be interpreted cautiously because lower-limb alignment parameters may vary according to age, sex, skeletal maturity, ethnicity, radiographic technique, and measurement method. AIS: adolescent idiopathic scoliosis; MAD: mechanical axis deviation; NSA: neck–shaft angle; aLDFA: anatomical lateral distal femoral angle; mLDFA: mechanical lateral distal femoral angle.

## Data Availability

The data supporting the findings of this study are available from the corresponding author upon reasonable request.
